# Post-COVID-19 Related Rhinocerebral Mucormycosis in Bulgaria: A Report of Three Cases

**DOI:** 10.7759/cureus.72434

**Published:** 2024-10-26

**Authors:** Kamelia Milcheva, Nikolay R Sapundzhiev, Radoslav Georgiev, Georgi S Stoyanov, Denis Niyazi

**Affiliations:** 1 Otolaryngology, Medical University of Varna, Varna, BGR; 2 Radiology, Medical University of Varna, Varna, BGR; 3 Pathology, Multiprofile Hospital for Active Treatment, Shumen, BGR; 4 Microbiology and Virology, Medical University of Varna, Varna, BGR

**Keywords:** bulgaria, covid-19, mucormycosis, rhino-orbital-cerebral mucormycosis, treatment

## Abstract

Rhinocerebral mucormycosis is a rare and rapidly progressive fungal infection caused by opportunist fungi from the Mucoraceae family, which are omnipresent in nature. Fungal sporangiospores discharged into the atmosphere could be inhaled or directly penetrate damaged skin and mucosa, and they strongly prefer angioinvasive spread. These relatively rare and opportunistic infections have spiked in recent years, particularly during the coronavirus disease identified in 2019 (COVID-19). Herein, we describe a series of three cases of rhino-orbital-cerebral mucormycosis (ROCM) during the second and third waves of COVID-19 in Bulgaria. The average age of the patients was 60.3 years (range 40-73, standard deviation ± 17.8). All of them contracted COVID-19 prior to developing ROCM and have a common comorbidity: type II diabetes mellitus. One of the cases was further complicated with a cerebral abscess as a result of ROCM. All patients underwent surgical treatments, and full recovery was achieved in two of the cases. In the third case, despite the extent of surgical and pharmaceutical treatment, ROCM progressed to a stable chronic disease.

## Introduction

”Fungi are the interface organisms between life and death.” With this quote, Paul Stamets starts his book “Mycelium Running" [1]. Rhino-orbit-cerebral mucormycosis (ROCM) is a sporadic and rapidly progressive fungal infection with a mortality rate over 50%, caused by opportunistic fungi belonging to the class Phygomycetes, subclass Zygomycetes, order Mucorales, family Mucoraceae that has a strong preference for angioinvasive spread [2]. They are found in decomposing organic materials and are ubiquitous. They are also thermotolerant. Most human infections are caused by direct injection into damaged skin or mucosa or by inhalation of fungal sporangiospores discharged into the atmosphere. Based on the anatomical localization, six types of mucormycosis could be established: rhino-orbital-cerebral, pulmonary, cutaneous, gastrointestinal, disseminated, and with an uncommon presentation [3]. Among them, ROCM is the most commonly occurring one [4].

The Leading International Fungal Education (LIFE) portal has estimated the global burden of serious fungal infections. According to their estimate before the coronavirus disease identified in the 2019 (COVID-19) pandemic, the annual prevalence of mucormycosis was estimated to be around 10,000 worldwide, barring cases from India, where the annual incidence is around 900,000 cases [3,5,6]. The estimated incidences per million in different regions are established as follows: Europe (from 0.2 cases in Denmark to 95 cases in Portugal), the United States of America (3.0 cases), Canada (1.2 cases), and Australia (0.6 cases). Prakash et al. estimated the pre-COVID-19 prevalence of mucormycosis to be 140 cases per million in India [3].

During the outbreak of the COVID-19 pandemic, Hoenig et al. identified 80 cases of COVID-19-associated mucormycosis between October 1, 2019, and April 12, 2021, including 29 (36%) unpublished cases [7]. Cases were reported from 18 countries, the majority from India (42), the United States of America (11), Pakistan (five), France (four), Iran (four), Mexico (four), and Russia (two). Single cases were reported from Austria, Bangladesh, Brazil, Chile, the Czech Republic, Germany, Italy, Kuwait, Lebanon, Turkey, and the United Kingdom [7].

Although the rise in ROCM cases has been noticed worldwide, Asia is experiencing the highest levels of it [8]. Immunosuppressed patients or those with poorly controlled diabetes are most prone to infection. The fungal spores invade the nasal mucosa and are not phagocytized as in immunocompetent individuals. They germinate, developing into angioinvasive hyphae that incite infarction of the nasal mucosa, resulting in "dry" gangrene findings. The initial presenting features of ROCM are nonspecific and frequently attributed to sinonasal or orbital pathologies. Angioinvasion helps dissemination to the target organs and provides a wide range of clinical manifestations.

## Case presentation

**Figure 1:**
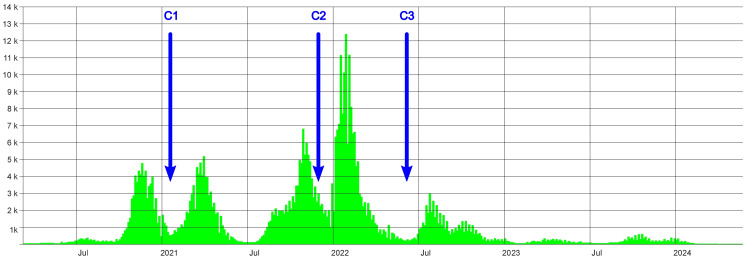
Incidence of COVID-19 cases and waves in Bulgaria Based on data from the COVID-19 Single Information Portal in Bulgaria. C1: presentation of the first case; C2: presentation of the second case; C3: presentation of the third case COVID-19: coronavirus disease identified in 2019

Herein, we present three cases of ROCM diagnosed and treated in a tertiary health care center in Varna, Bulgaria. The cases were presented between January 2021 and May 2022, during the peaks of the second and third waves of COVID-19 in Bulgaria (Figure 1). 

Case 1

**Figure 2:**
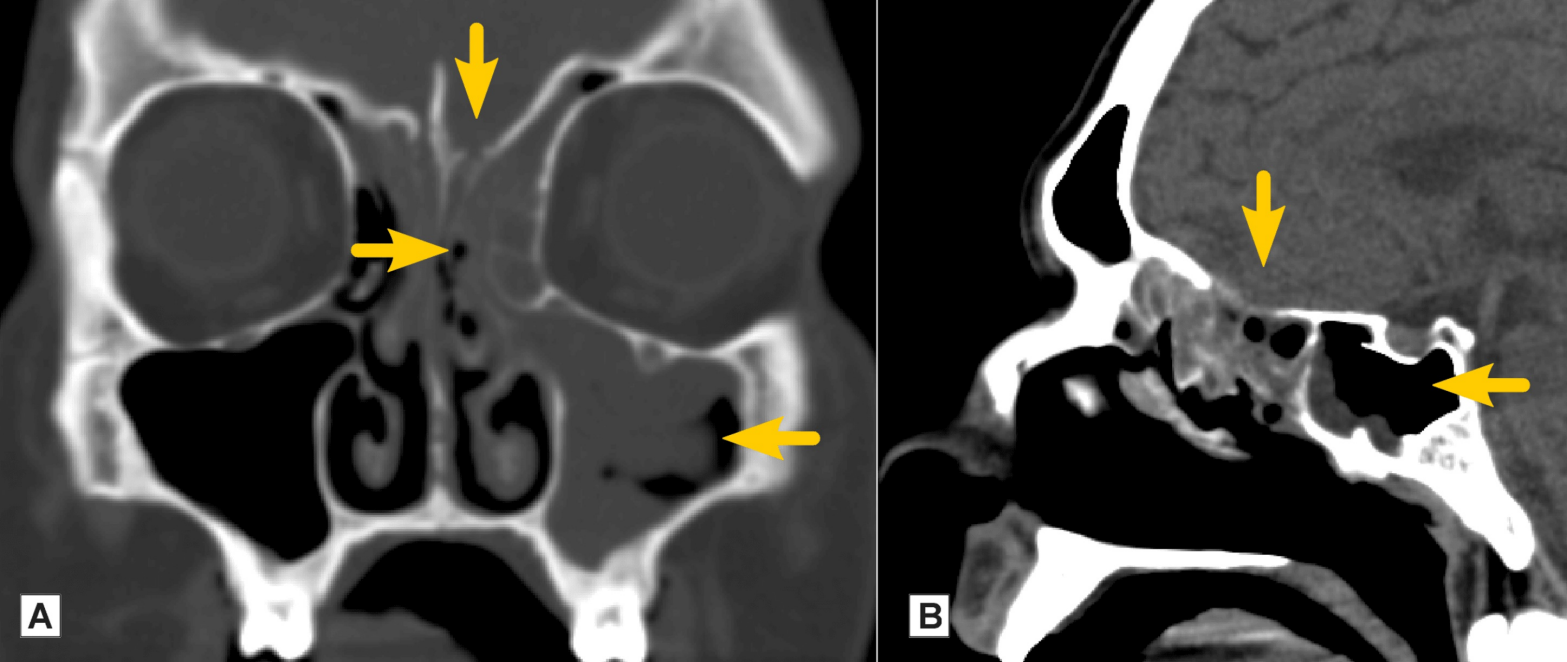
Native CT evaluation (Case 1) A: coronal plane showing erosion of lamina cribrosa and left-sided pansinusitis (arrows); B: sagittal plane showing routh to the anterior cranial fossa and sphenoidal sinusitis (arrows)

A 40-year-old male presented to our institution in January 2021. He was with a two-year-long duration of type II diabetes mellitus. His treatment included perioral Janumet 50/850 mg twice daily, which provided good control of the disease. Previous medical history was significant for COVID-19 infection two months prior, with bilateral pneumonia treated within a hospital institution with a combination of levofloxacin, ceftriaxone, meropenem, fluconazole, and dexamethasone and supported with supplemental oxygen therapy by face mask for two weeks. He was not vaccinated against coronavirus infection. Current complaints were of rhinorrhoea and severe paroxysmal headache. The patient described throbbing and piercing pain on the left side of the face and left orbit for the previous month and double vision developing for the previous ten days. The patient was admitted to the neurology department, and Staphylococcus aureus was isolated from nasal discharge. A computer tomography (CT) scan demonstrated inflammatory changes in the soft tissues within the left maxillary sinus, sphenoid sinus, and many ethmoid cells. There was an osteolysis of lamina cribrosa on the left side (Figure 2).

**Figure 3:**
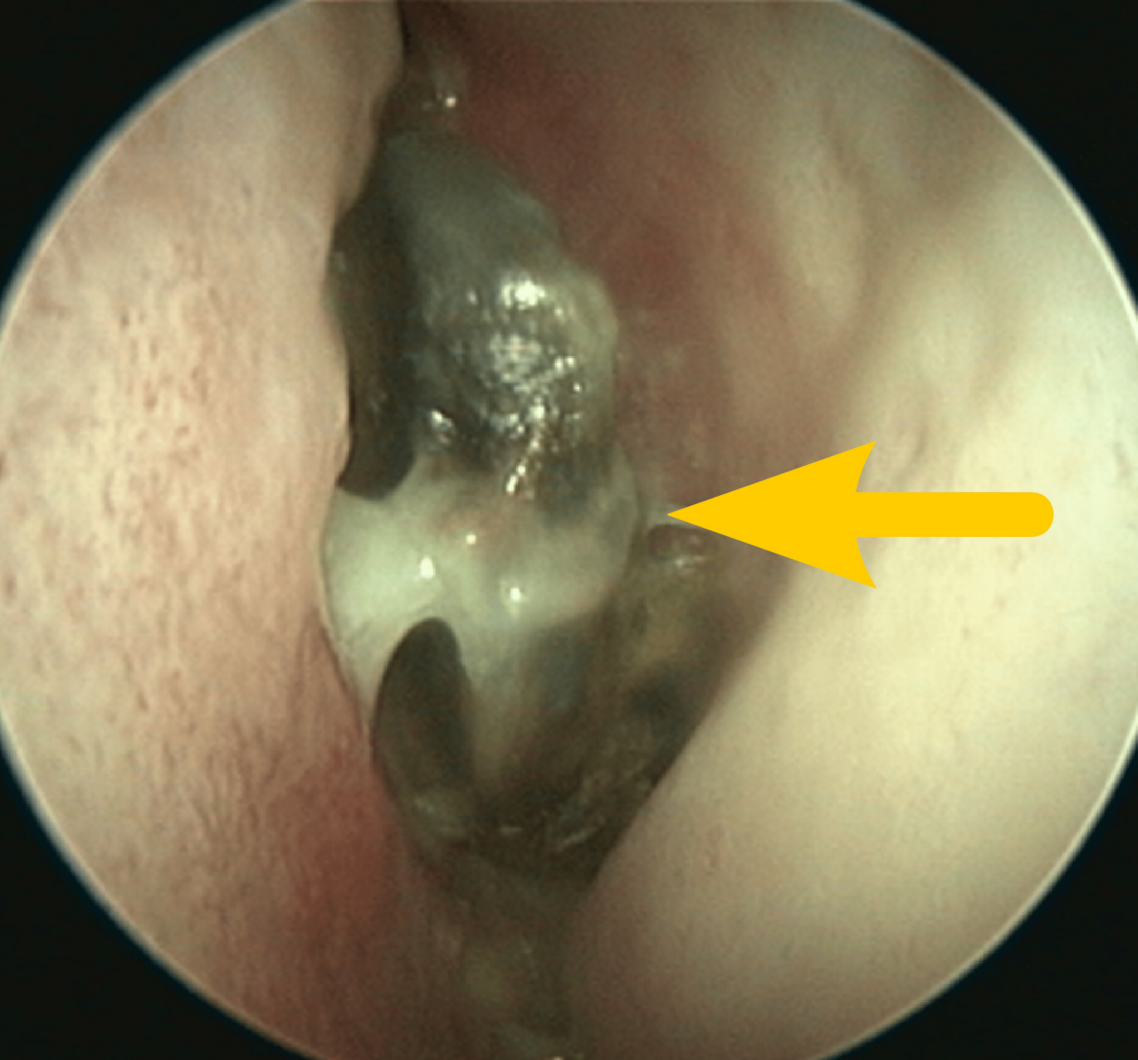
Endoscopic view of the mummified left medial turbinate (arrow) (Case 1)

Rhinoscopy showed an atypical black mummified middle nasal concha on the left side with normal surrounding mucosae and structures (Figure 3).

**Figure 4:**
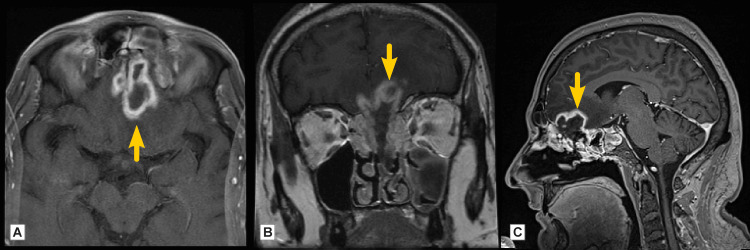
MRI post-contrast evaluation (Case 1) A: axial, B: coronal, and C: sagittal plane showing cerebral abscess (arrows) with perifocal edema and left-sided pansinusitis

Further imaging diagnostics included a secondary CT scan and magnetic resonance imaging (MRI), which showed signs of chronic pansinusitis predominantly on the left side and a bilateral frontal lobe brain abscess (Figure 4).

**Figure 5:**
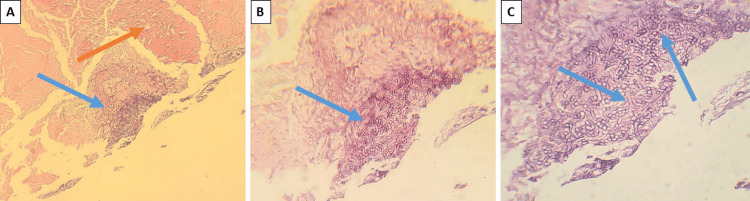
Histopathological findings (Case 1) A: necrotic tissue (orange arrow) and perivascular hyphae (blue arrow), H&E stain, original magnification 40x; B: broad pauciseptate hyphae (arrow), H&E stain, original magnification 100x; C: pauciseptate hyphae with irregular 90-degree branching (arrows), H&E stain, original magnification

Intraoperatively, the left nasal cavity was filled with necrotic matter, and necrosis also affected the medial nasal concha and the posterior part of the bony nasal septum. Left middle turbinectomy, ethmoidectomy, and nasal debridement were performed. Additionally, there was a route through the cribriform plate into the skull with the involvement of the brain's frontal lobes. Drainage of the abscess was achieved through the melted lamina cribrosa. The diseased tissues were very well delineated from the adjacent normal tissues, with minor signs of inflammation and almost no bleeding. Histopathology showed inflamed granulation tissue with mixed inflammatory cells, including neutrophils, plasma cells, macrophages, and lymphocytes (Figure 5).

Tissue cultures grew Staphylococcus aureus. Subsequent follow-up demonstrated good wound healing. No more necrotic tissue occurred in the nasal cavity, and after a smooth postoperative period, the patient was discharged from the hospital. No antifungal treatment was provided as the diagnosis was not clear. Only retrospectively, mucormycosis was suspected. A revision of histological samples with additional special stains revealed branched hyphae with wide-angle (90°) bifurcations, typical for Mucorales.

Case 2

**Figure 6:**
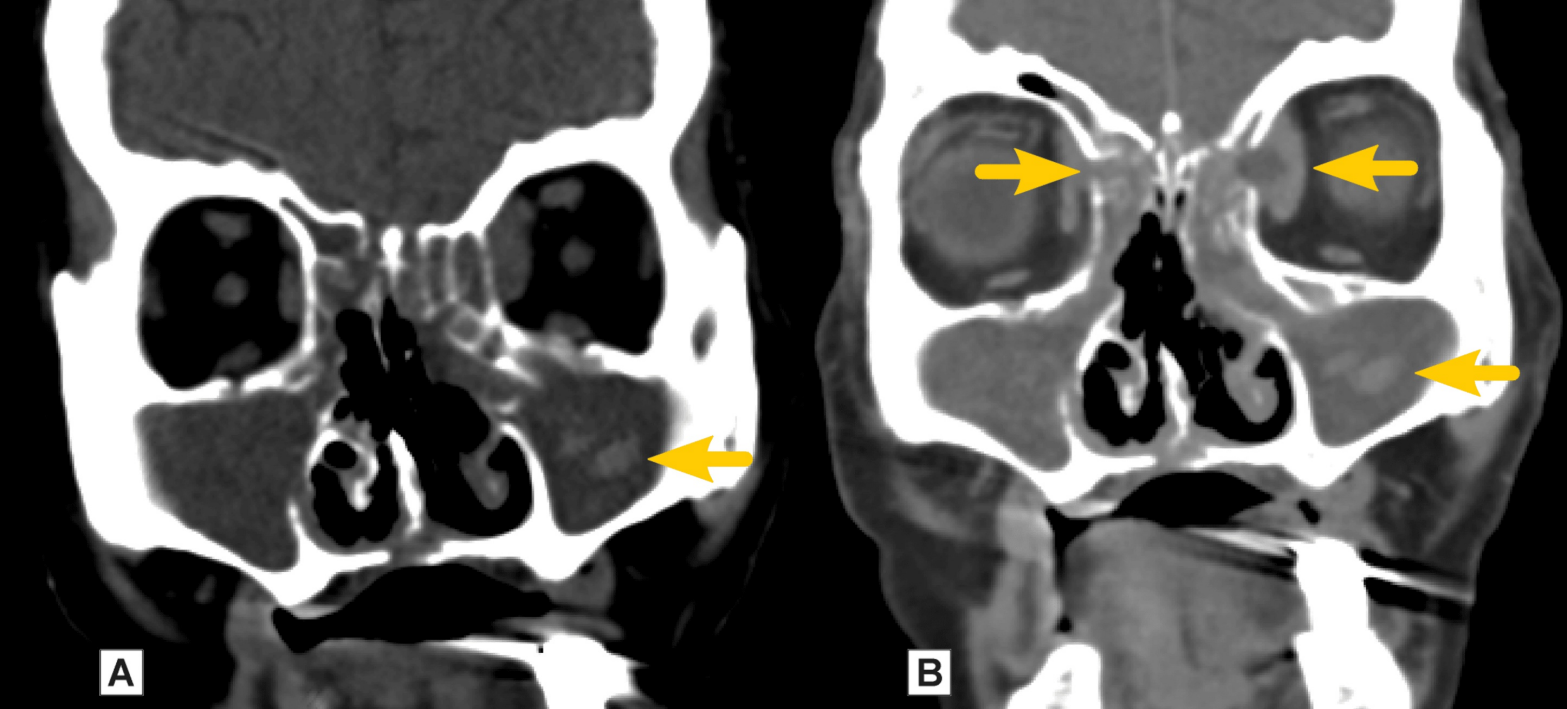
CT findings (Case 2) A: native CT in the coronal plane showing hyperdense regions in the maxillary sinuses (arrow); B: postcontrast CT showing no significant contrast enhancement of the regions and thinning and osteolysis of the medial wall of the right orbit with some fluid collection (arrow)

A 68-year-old female with a past medical history significant for type II diabetes mellitus presented in December 2021 with nasal breathing difficulty, purulent nasal discharge, severe pain in the nose, redness and sagging of the external nose, swelling, and redness around the left eye. Relevant medical history revealed a five-year-long duration of diabetes with poor control and was treated with metformin 1000 mg twice daily. Three months before presentation, the patient had been admitted twice for COVID-19 with bilateral pneumonia, hyperglycemia over 50.0 AU/ml, renal failure, and had been treated with a combination of cefoperazone, levofloxacin, amoxicillin/clavulanic acid, meropenem, and corticosteroids. No supplemental oxygen therapy was needed due to COVID-19. She had had several nasopharyngeal swabs for COVID-19 on different occasions. She was not vaccinated against coronavirus infection. Nasal endoscopy showed an abundance of crusts in the nose. A CT scan demonstrated chronic pansinusitis and osseous defects in the wall of ethmoid cells, medial turbinates of both sides, inferior turbinates, and medial wall of the maxillary sinuses, lamina papyracea, nasal septum, and hard palate on the midline (Figure 6).

There was a small abscess between the ethmoid and the rectus medialis muscle in the left orbit and a thickness of about 3 mm in the soft tissues in the medial wall of the right orbit.

Endoscopic surgical treatment with aggressive debridement of the affected tissue under general anesthesia was undertaken. Intraoperatively, the nasal septum was involved with a subtotal defect and necrosis of the inferior right turbinate, and both middle turbinates were found. Ethmoidectomy and endoscopic medial maxillectomy were also performed. Sequestrectomy only partially involved the lamina cribrosa and vomer.

**Figure 7:**
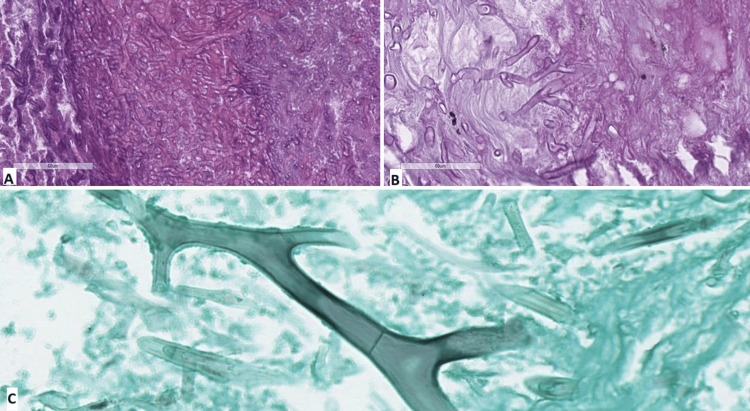
Histopathological features of mucormycosis (Case 2) A: fungal microorganisms are seen on hematoxylin and eosin, original magnification x400; B: fungal microorganism septae and branching seen of PAS-stain, original magnification x400; C: fungal microorganism septae and branching, Grocot stain, original magnification x400

Microbiology showed Enterobacter cloacae, which is sensitive to amikacin, cefepime, ciprofloxacin, levofloxacin, imipenem, and meropenem. Histopathological investigation of the tissue confirmed Mucormycosis with angioinvasion based on the typical appearance of the hyphae (Figure 7).

Postoperative nasal irrigations and local measures following surgery had variable effects due to the development of a fistulization on the left side of the root of the nose to the skin. Antifungal treatment was not administered because of a nationwide temporal shortage of amphotericin B. The patient was lost to follow-up. In 2024 (three and a half years after the discharge from our department), the patient presented to outpatient care and was in good general condition. Her current complaints were of heavily impaired nasal breathing and unpleasant odor. The external nose was badly deformed with a minor whole-thickness defect of the skin, measuring a few millimeters.

Case 3

**Figure 8:**
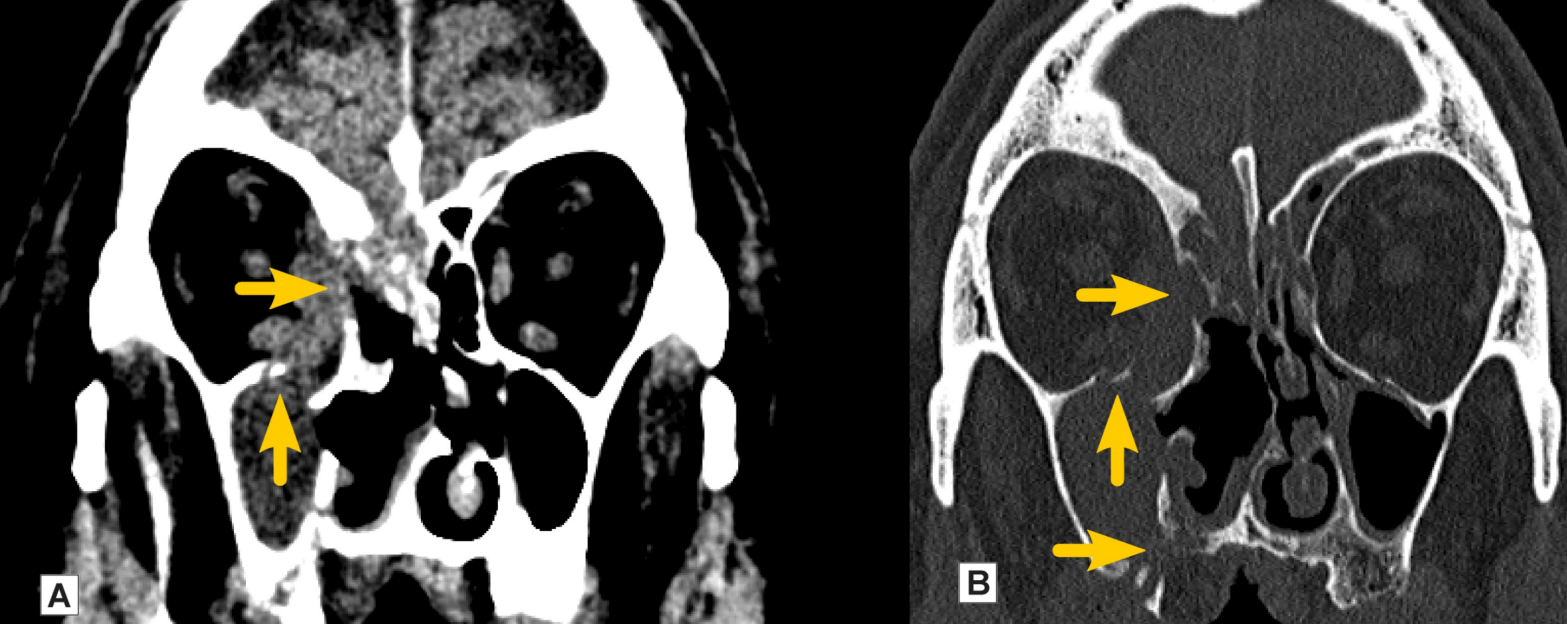
Native CT findings (Case 3) Native CT (A) and native CT bone window (B): filled with liquid density content of the right maxillary sinus with osteolysis of the right maxillary sinus's superior wall (arrow), the right orbit's medial wall (arrow), and oroantral fistula (arrow)

A 73-year-old male was admitted in April 2022 to the department with a headache and recurrent nasal bleeding. He was not vaccinated against coronavirus infection. Previous medical history was significant for COVID-19 three months prior. The patient, who had suffered from well-controlled type II diabetes mellitus, was treated with metformin 1000 three times daily. Clinical findings and imaging showed the destruction of the right maxillary sinus medial, inferior, and posterior walls and the involvement and partial destruction of the pterygopalatine fossa and the pterygoid processes. The ethmoidal labyrinth on the right side and the frontal sinus were involved similarly. Inflammation caused erosion of the medial wall of the orbit and the development of a subperiosteal abscess. The medial rectus and oblique muscles were affected. There were also signs of propagation to the anterior cranial fossa with infiltration of the meninges on the right side of the crista galli. Communication between the nasal and oral cavity with the oroantral fistula was detected (Figure 8).

The patient underwent endoscopic sinus surgery under general anesthesia. All sequestered tissues were removed. Again, a clear delineation of the crusts and necrotic fragments from the surrounding minimally altered normal tissues was noted. The debridement resulted in a large septal defect. Histology confirmed the diagnosis of Mucormycosis.

**Figure 9:**
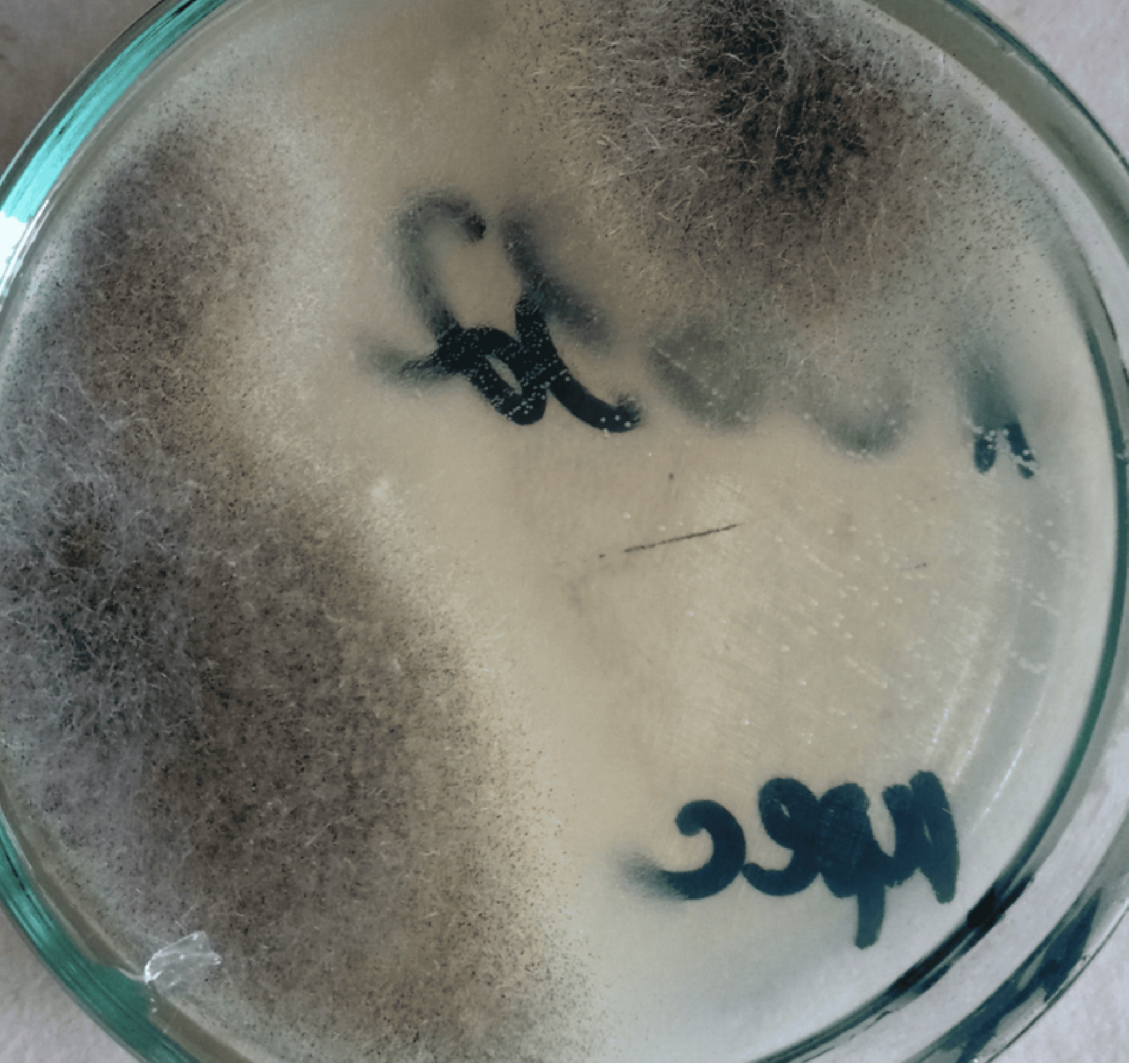
Growth of grey or black cotton candy-like colonies of Mucorales on Sabouraud agar (Case 3)

Fungal culture samples obtained from clinically active parts of the lesion during surgery resulted in grey and black cotton candy-like colonies on Sabouraud agar (Figure 9).

**Figure 10:**
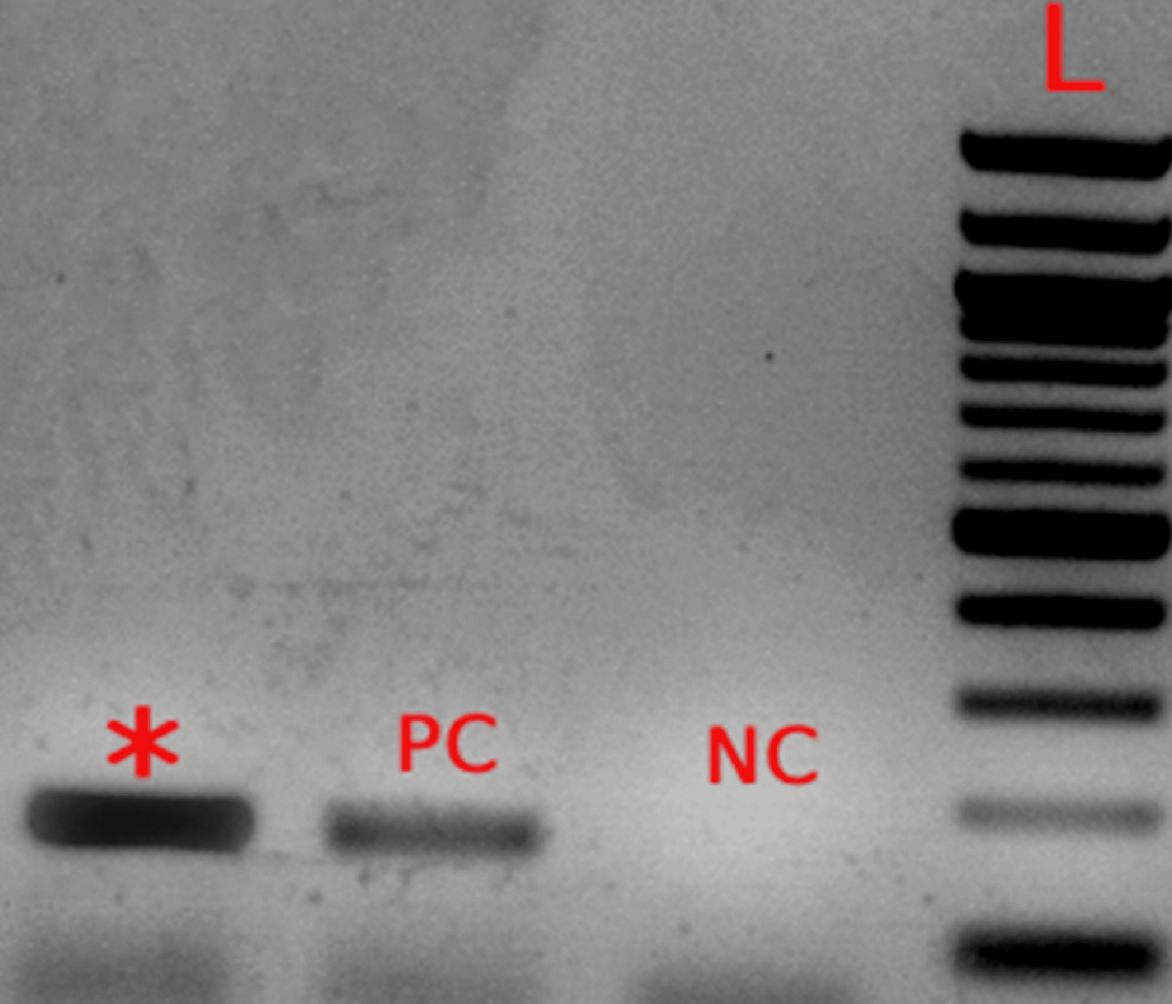
PCR 18S rRNA gene in Mucorales spp. (Case 3) * sample; PC: positive control; NC: negative control; L: DNA marker 100 bp; PCR: polymerase chain reaction; RNA: ribonucleic acid

In addition, a polymerase chain reaction (PCR) test of the removed nasal tissue was positive for Mucorales (Figure 10).

In the early postoperative period, endoscopy showed normal tissue recovery with no residual crusts, smooth mucosa, and no signs of inflammation. Without any other clinical signs of local or generalized involvement, no antifungal treatment was administered.

## Discussion

We encountered three cases (two male and one female) with ROCM in our otorhinolaryngological (ORL) department during the second and third waves of COVID-19 in Bulgaria. The average age of the patients was 60.3 years (range 40-73, standard deviation ± 17.8). All of them had a recent medical history of COVID-19 before admission, and all of them had a common comorbidity: type II diabetes mellitus. None of them was vaccinated against coronavirus infection.

Epidemiology

In the pre-COVID-19 period, the incidence of mucormycosis in India (0.14 per 1000 capita) was 80 times higher than what was reported in the world (0.005-1.7 per million capita) [9]. A surge in cases of post-COVID-19 ROCM has been reported over the past few years. A review of existing literature indicates that 81% of instances of COVID-19-associated ROCM were related to India [9]. Small case series or reported cases came from other countries like Turkey, Egypt, and Iran. This indicates that mucormycosis was an endemic disease in India before and during the COVID-19 pandemic [9].

Nevertheless, an unseen or undiagnosed condition for us, ROCM, was recognized in three cases during the COVID-19 pandemic. One probable explanation for this increased incidence of COVID-19-related ROCM in our department in Bulgaria may be the huge number of nasal tests during the pandemic. According to the National Health Information System (NHIS), 11,650,230 tests were done in Bulgaria (16.03.2024) [10]. With a population of 6.4 million and varying indications and target groups, a gross estimation can be made that all eligible for testing persons should have had three tests on average. Nasal sampling may be the most frequently performed ear-nose-throat (ENT) manipulation in Bulgaria due to the COVID-19 pandemic [11]. All three patients in our series had had more than three tests, as they were hospitalized for COVID-19 and must have gone through screening, diagnostic, discharge, and follow-up tests. Minimal traumatic injury to the nasal mucosa may be responsible for creating a gate for the inoculation of the ubiquitous Mucoraceae fungi. Although it is hard to track backward and avoid over-testing, nosocomial transmission cannot be ruled out during that time when specimen collection swabs came from atypical suppliers and were not always available [12].

Fungal sporangiospores inhaled into the nasal cavity and paranasal sinuses can cause ROCM to develop. At that point, the infection could spread quickly to nearby tissues. As soon as it germinates, the invasive fungus can invade in different directions, including the ethmoid, the sphenoid sinus, the orbits, the cavernous sinus, the scull, the brain through the orbit or the cribriform plate, and the palate.

Among the listed potential risk factors were uncontrolled diabetes, iron overload, corticosteroid medication, chemotherapy, stem cell and solid organ transplantation, malignancy, and immunodeficiency. New risk factors like chronic renal failure and tuberculosis have been described recently [3]. While pulmonary mucormycosis is more common in people with hematological malignancies and transplant recipients, ROCM is more common in people with diabetes mellitus. It has been established that diabetes is a separate risk factor for both severe COVID-19 and ROCM, particularly when it is poorly managed or accompanied by diabetic ketoacidosis [13]. Acidosis increases free iron levels, promoting invasive fungal growth. Endothelial damage, thrombosis, lymphopenia, and reduction in cluster of differentiation (CD)4+ and CD8+ T-cell levels predispose to secondary or opportunistic fungal infection [13].

Diagnostics

The literature lists a wide range of warning signs and symptoms of ROCM, which include a foul smell, blood-tinged, purulent, or black nasal discharge, epistaxis, nasal mucosal erythema, eyelid, periocular or facial edema or discoloration, orbital pain, paranasal sinus or dental pain, facial pain, headache, proptosis, sudden vision loss, sudden ptosis, facial paraesthesia, anesthesia, diplopia with ocular motility restriction, facial palsy, fever, paralysis, and focal seizures [14]. So, our patients presented with well-localized symptoms, mainly impaired nasal breathing, fault odor, and, in one case, pain in the nasal/facial region. All our patients had no or minimal general symptoms.

Microscopy and culture are essential for the diagnosis of ROCM. Molecular tests can be employed to identify or detect Mucormycetes, and they might be suggested as useful supplementary instruments to support traditional diagnostic techniques [15]. A quick diagnosis can be made using direct microscopy of the deep or endoscopy-guided nasal swab, paranasal sinus, or orbital tissue using calcofluor white and a potassium hydroxide mount. Direct microscopy has about 90% sensitivity [14].

The most widely used in histopathology are hematoxylin-eosin, periodic acid-Schiff, and Grocott-Gomori's methenamine-silver. Samples taken from the paranasal sinus mucosa, orbital tissue, and nasal mucosa should be referred to rapid diagnostic tests like squash and imprint and processed for routinely fixed sections. Mucorales hyphae are non-septate or pauci-septate, with a variable width ranging from 6 to 25 μm with an uneven, ribbon-like appearance. There are a variety of branching angles, most commonly broad bifurcations around 90° [16]. Diagnosis of almost 80% of samples with potential ROCM is provided by histopathology [14].

The predominant inflammatory process in tissue histology is neutrophilic or granulomatous [17]. In the samples of immunocompromised patients, inflammation is usually absent [18]. Typical manifestations include prominent infarcts and angioinvasion, which cause tissue necrosis and vascular thrombosis [19]. Furthermore, compared to non-neutropenic patients, neutropenic patients exhibit a more widespread angioinvasion [20].

Cultures of the paranasal sinus, orbital tissue, or deep or endoscopy-guided nasal swabs reveal the fast growth of fluffy white, grey, or brown colonies that resemble cotton candy. On most fungal cultures, including potato dextrose agar and Sabouraud agar, incubated at 25°C to 30°C, all Mucorales develop in three to seven days. Fungal cultures are positive in only 50% of samples taken from clinically active portions of the lesion (not from extensively necrotic tissue), even when fungal hyphae are visible in histopathologic investigation [14].

Mucormycetes can be found or identified by molecular tests using samples from nasal swabs, paranasal sinus or orbital tissue, cultures, or blood. Molecular-based tests include sequencing of specific gene areas, restriction fragment length polymorphism analyses (RFLP), traditional PCR, and melt curve analysis of PCR products [14]. PCR and in situ hybridization provide an alternative for early diagnosis essential for patients' survival. When accessible, molecular assays can be employed for diagnosis confirmation with a sensitivity of approximately 75% [14].

Imaging techniques like CT and MRI take part in the diagnosis of ROCM. They are useful for determining complications and the extent of the disease. In cases of suspected orbital or cerebral involvement, contrast-enhanced MRI studies are preferred over CT scans. It helps identify orbital cellulitis, internal carotid artery thrombosis, and cavernous sinus thrombosis. MRI is the gold standard, while CT is often used in conjunction. An early sign is a thickening of the nasal and paranasal sinus mucosa with irregular patchy enhancement. Fluid level and partial or total opacification are nonspecific and usually indicate advanced involvement of the sinuses. An early indicator of orbital invasion is the thickening of the medial rectus muscle. Advanced disease is demonstrated by an enhancement in the orbital fat tissue or orbital apex, superior and inferior orbital fissures, and bone erosion of the paranasal sinus walls and orbit. For ROCM, the lack of involvement of the paranasal sinuses has a substantial negative predictive value [14].

In our series, the diagnosis was suspected on endoscopy and confirmed by histopathology. All other tests were supplemental. The imaging modalities are aimed at estimating the spread of the disease and planning the surgical debridement and the follow-up, especially in intracranial or orbital involvement. With typical endoscopic appearance, the diagnosis is easy to suspect for otorhinolaryngologists who are aware of this condition. However, with a complete lack of pre-COVID experience with ROCM in our geographic region and department, we only diagnosed the first case retrospectively. With raised awareness of ROCM, we have seen no further cases after the last peak of COVID-19 in the summer of 2022. This may be seen as proof of the relation between both infections or the repeated trauma from nasal swabs and mucormycosis.

Treatment

A multimodal approach is necessary for the successful management of ROCM. It includes surgical debridement of all infected tissues, administration of active antifungal agents, various adjunctive therapies, and reversal or cessation of underlying predisposing risk factors.

Regarding the severity of acidosis, using sodium bicarbonate in conjunction with insulin to reverse ketoacidosis and improve the prognosis of the condition by compromising Mucorales' ability to spread in host tissues.

Amphotericin B is the most active drug. Posaconazole and isavuconazole are also beneficial. Since antifungal medications cannot penetrate non-vascularized necrotic tissues, early surgery is essential to lessen the fungal burden.

Surgery needs to be forceful. It is important to remove the necrotic and surrounding healthy-looking tissues. Patients with mucormycosis were shown to have a statistically significant increase in survival after surgical debridement.

The endoscopic approach is much better than open surgery and could provide an excellent outcome. In our experience, early (case one) and late (case three), extensive debridement sufficed in two patients with purely intracavitary and intracranial involvement. In the second case, we may speculate that the chronic stable local disease is due to insufficient debridement during our first intervention; "radical" surgery in this patient would have meant resection of the external nose. Still, this is very difficult to judge, as the patient was initially lost to follow-up and systemic antimycotic treatment.

## Conclusions

During the second and third waves of the COVID-19 pandemic, we encountered three ROCM cases that had never been diagnosed and were unknown to us. The number of ROCMs reported worldwide faded after the COVID-19 pandemic. Our local and global data again suggest the relation between ROCM and coronavirus infection or ROCM and an overwhelming number of nasal swabs. Despite the etiologic link, the predominance of diabetes as a predisposing risk factor is clear. Whether the cases of ROCM will return to their usual pre-pandemic levels remains to be seen.

Endoscopic examination plays a major role in diagnosing this rare disease. In any atypical dry necrotic tissue or crust formation in the nasal cavity, mucormycosis could be suspected, and histological investigation is required to confine or exclude it. Early surgical debridement takes the most important place in the treatment of ROCM and could be successful even without administering antifungal medications. The endoscopic surgical approach should be the first option in cases with no external nose/skin involvement.
